# First Clinical Results of a New Generation of Ablative Solid-State Lasers

**DOI:** 10.3390/jcm12020731

**Published:** 2023-01-16

**Authors:** Bojan Pajic, Brigitte Pajic-Eggspuehler, Zeljka Cvejic, Christian Rathjen, Viktor Ruff

**Affiliations:** 1 Eye Clinic Orasis, Swiss Eye Research Foundation, 5734 Reinach, Switzerland; 2 Department of Physics, Faculty of Sciences, University of Novi Sad, Trg Dositeja Obradovica 4, 21000 Novi Sad, Serbia; 3 Division of Ophthalmology, Department of Clinical Neurosciences, Geneva University Hospitals, 1205 Geneva, Switzerland; 4 Faculty of Medicine, University of Geneva, 1205 Geneva, Switzerland; 5 Faculty of Medicine of the Military Medical Academy, University of Defense, 11000 Belgrade, Serbia; 6 Ziemer Ophthalmic Systems, 2562 Port, Switzerland

**Keywords:** solid-state laser, ablation laser, deep-UV, refractive surgery

## Abstract

In the early 2000s, solid-state lasers emerged as an alternative technology to excimer systems in refractive surgery. Despite some technological limits at the time, good clinical results could be achieved with solid-state laser systems. This prospective case series reports clinical outcomes of five eyes treated with a newly developed solid-state laser system (AquariuZ) in three patients. Patients underwent preoperative examination, including corneal topo-and tomography, aberrometry, and confocal microscopy. All patients received a femtosecond LASIK with the Ziemer LDV Z8, a refractive treatment with the AquariuZ solid-state ablation laser, and were then followed up for a period of up to 12 months. The applied aspheric optimized profiles did not induce higher-order aberrations nor spherical aberration in any of these operated subjects. No eye lost BCVA lines throughout the duration of the follow-up. Six months after surgery, the safety index of patient 1 was 5, and for patients 2 and 3, it equaled 1. Confocal laser microscopy imaging findings were comparable to those seen typically for excimer lasers. The obtained results are encouraging and confirm that the new solid-state laser system is safe.

## 1. Introduction

Shortly after the invention of lasers, the concept of an excimer laser was proposed in 1960 by Fritz Houtermans [1]. After some basic research, the first excimer lasers became commercially available in the late 1970s [2]. Srinivasan discovered the possibility of ablation of materials under intense illumination using ultraviolet laser pulses [3,4]. In 1983, in collaboration with S. Trokel, the concept of ablative laser vision correction was developed [5]. There appear to be early records of excimer treatment of human eyes dating back to the years 1987 and 1988 [6,7]. Clinical prospects combined with mature laser technology led to rapid development of laser vision correction applications. PRK (Photorefractive Keratectomy) was first introduced in the early 1990s, and FDA-approved in 1995. LASIK (Laser-assisted in-situ keratomileusis) followed shortly after and has quickly become the gold standard of laser vision correction available today.

At the same time as industrial excimer lasers for ophthalmology became available, a new class of laser crystals was introduced. With these crystals, an array of frequency conversion schemes was possible, spanning a broad wavelength range from deep UV to mid-infrared [8].

This led almost immediately to the first attempts to build solid-state deep UV laser sources. LaserSight developed the first system called LaserHarmonics [9,10], whilst another one, called LightBlade, was developed by Novatec [11]. Although their first clinical outcomes were promising, the technology had to face severe competition from excimer laser systems. Excimer lasers had the advantage of providing high pulse energies that enabled large beam ablation for refractive surgery purposes already at low-repetition rates. The use of a solid-state laser running at 10 Hz indicated that increasing the repetition rate was necessary in order to meet market expectations related to ablation speed at that time [9]. Compared to excimer sources, which were available as industrial components with a sufficiently high output, the ability of solid-state laser sources to produce high pulse energies in order to achieve reasonable treatment times was limited at that time [12].

In a review paper published in 1995 [13], an overview of contemporarily available refractive surgical lasers, including excimer laser systems and solid-state laser systems under development spanning a wavelength range from 205 to 220 nm, was provided. A comparison of their respective performance parameters revealed that solid-state lasers did not deliver tens of millijoules of pulse energy required to achieve state-of-the-art large beam ablation for clinical use. At that time, high-repetition-rate lasers combined with scanning spot technology and eye tracking were proposed for future refractive lasers [13]. Such technology and systems became only available in the initial years of the current millennium [14].

First clinical results obtained with ablative solid-state lasers were simultaneously published in 2004 by two independent research groups, i.e., Anderson and co-workers utilizing a CustomVis Pulzar Z1 (Balcatta, Australia) and Roszkowska et al. with the LaserSoft Katana (Kleinmachnow, Germany) [15,16]. Later, Tikhov and co-workers described a solid-state laser system, OLIMP (Yaroslavl, Russian Federation), introduced in 2009 [17,18].

In these early studies, the researchers pointed out the potential benefits of solid-state ablation laser technology. They reported excellent beam quality along with stable energy output, while producing smooth ablation surfaces [15,16,17,18]. Furthermore, based on the work of Hale and Querry on extinction coefficients of radiation over a large area of the electromagnetic spectrum, Dair and co-workers found less absorption of up to two orders of magnitude for solid-state lasers in Balanced Salt Solution (BSS) and 0.9% Sodium Chloride solution compared to an excimer system [19,20]. While for excimer lasers emitting at 193 nm, tissue hydration control was found to be crucial to achieve target ablation [21,22,23], solid-state ablation lasers emitting at longer wavelengths would offer the benefits of being less dependent on the hydration state of the cornea and the humidity of the surgical environment, as well as not using toxic gas and operating silently [24,25,26,27,28].

The above-stated advantages, although proven, required a company with no legacy in excimer laser products to make a new start and conceive a laser system with technology not available in the early days of solid-state lasers. This prompted Ziemer Ophthalmic Systems to choose solid-state over excimer laser technology for the development of a new ablation laser.

In this case series, we report the clinical outcomes of three patients receiving LASIK with a new solid-state UV laser (AquariuZ, Ziemer Ophthalmic Systems AG, Port, Switzerland) developed in compliance with the new European Medical Device Regulations (MDR).

## 2. Materials and Methods

This was a prospective observational case series including 5 eyes of 3 patients conducted in accordance with the Declaration of Helsinki and all applicable regulatory requirements. All patients provided their written consent for the publication of their surgery-related data. Patients were included according to the device’s approved indications for use. All surgical procedures were performed by the same surgeon (B.P.).

A detailed ophthalmological status was obtained in all patients, including manifest refraction, cycloplegic refraction, UDVA, CDVA, slit lamp microscopy of the anterior segment, dilated fundoscopy, and ocular pressure measurement. Topography was obtained using the Galilei system (GALILEI G2, Ziemer Ophthalmic Systems, Switzerland) and aberrometry using Zywave (Bausch & Lomb, Rochester, NY, USA). All patients underwent confocal laser microscopy (HRT3 RCM, Heidelberg Engineering, Germany) of the interface during the postoperative period. Preoperatively, no patient was found to have a dry eye syndrome.

**Figure 1:**
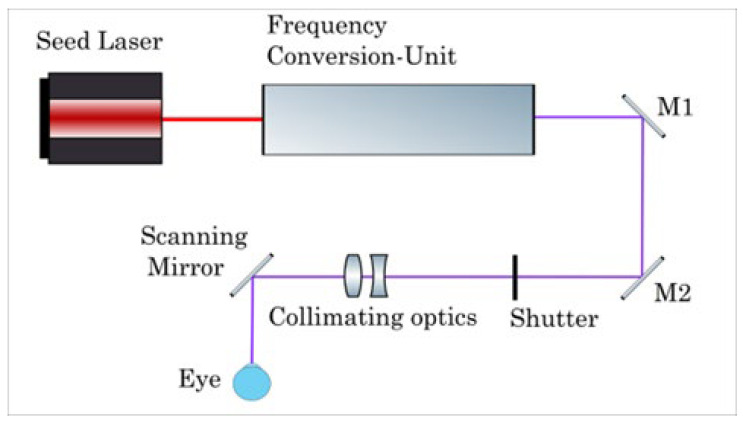
Scheme of the laser source and delivery system used in the AquariuZ system.

**Figure 2:**
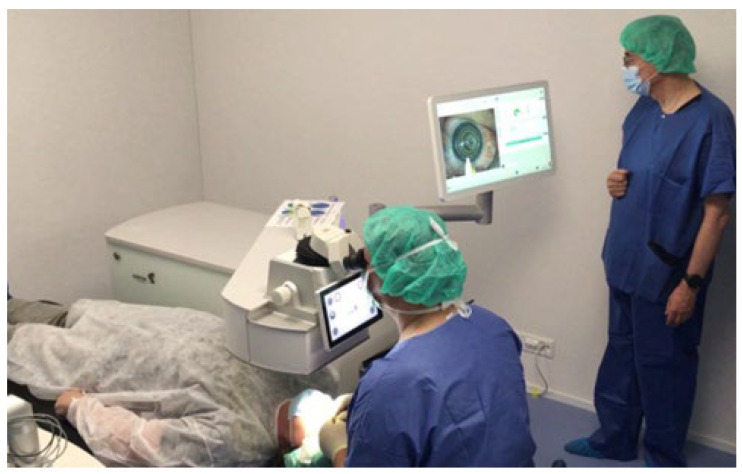
Footage of the AquariuZ in a clinical setting during a refractive procedure.

All patients underwent refractive surgery using the AquariuZ solid-state laser (Ziemer Ophthalmic Systems AG, Port, Switzerland). LASIK flaps were created with the FEMTO LDV Z8 femtosecond laser platform (Ziemer Ophthalmic Systems AG, Port, Switzerland) with a superior hinge and a target flap thickness of 110 µm. Ablations were performed with the AquariuZ solid-state UV laser, which obtained European conformity in March 2020. This laser system produces nanosecond UV pulses in the wavelength range of 205–215 nm by frequency conversion of an infrared (IR) seed laser. The output repetition rate is up to 500 Hz. It comprises a 6-dimensional eye-tracking system (including XY, Gaze, and Z-tracking) and optimized aspheric profiles, while using a small (<1 mm) spot. Figure 1 shows the beam path scheme of the AquariuZ, while Figure 2 shows a typical configuration during a refractive surgery with the AquariuZ, comprising a patient, surgeon, and assistant.

**Figure 3:**
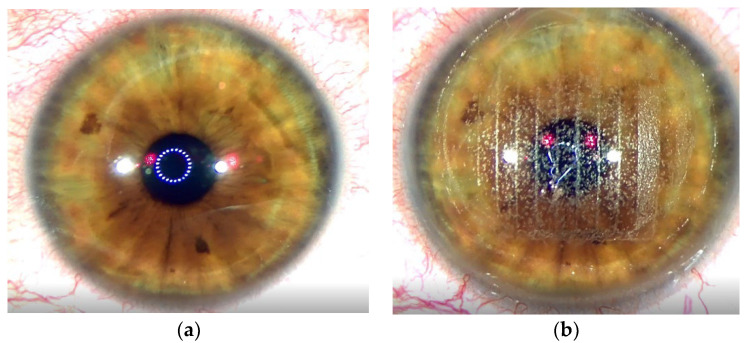

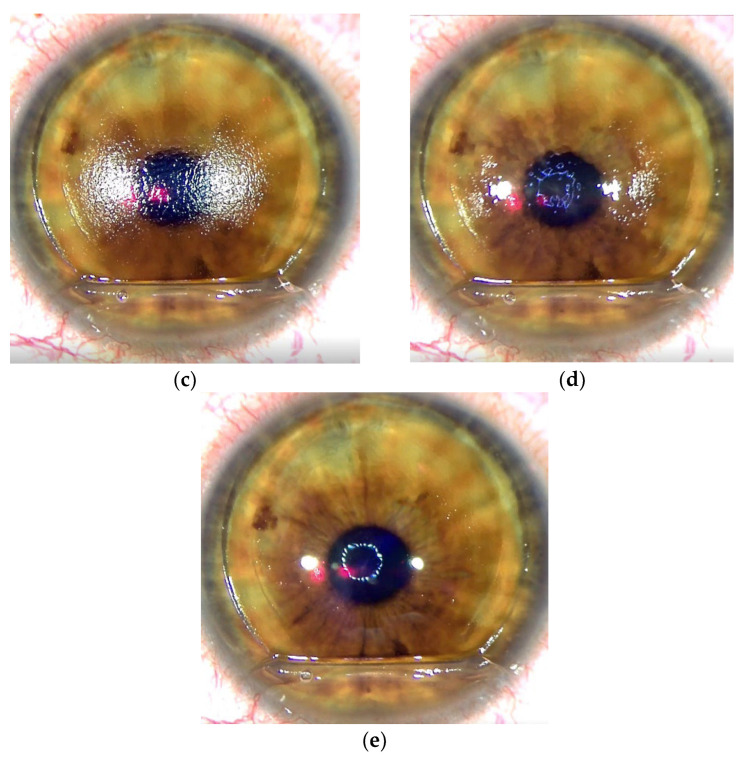
(**a**) Eye condition before treatment, (**b**) situation after femtosecond laser incision, (**c**) dry stroma bed before laser application, (**d**) fluid builds up on the stroma during laser treatment, (**e**) stroma bed covered with fluid after treatment.

The ablative solid-state laser has different treatment dynamics than the excimer laser due to the different laser wavelength applied. Figure 3a shows the condition of the cornea before treatment. Figure 3b depicts the status after the femtosecond laser flap creation. Figure 3c illustrates a dry stromal bed once the flap has been lifted. The ablative solid-state laser is then applied. Figure 3d shows the stroma bed after the application of the first pulses with Aquariuz. At the end of the treatment, the stroma is covered with a liquid film suggesting that the stroma stayed wet during the whole process (Figure 3e). The latter observation differs significantly from that characteristic of excimer lasers, where water is evaporated in the process leaving back an opaque surface in the ablated area.

In all patients, Tobradex (Alcon Laboratories, Inc., Fort Worth, TX, USA) was applied postoperatively three times daily for a total of seven days. In addition, topical hyaluronic acid 0.15% was applied three times daily for a total of at least one month.

## 3. Results

Within the scope of the study, a total of five (*n* = 5) eyes were treated in three (*n* = 3) patients. The mean age of patients was 37 ± 17 years. Preoperative corrected distance visual acuity (CDVA) was L (left eye L) 0.01 in patient 1, OD (right eye R) 1.0 and L 1.0 in patient 2, and R 1.0 and L 1.0 in patient 3 (Table 1). Preoperative Manifest Refraction Spherical Equivalent (MRSE) was L −7.00 D in patient 1, R 0.00 D and L −1.00 D in patient 2 and R −1.00 D and L −1.50 D in patient 3 (Table 2).

In patient 1, diagnosed with severe amblyopia, the preoperative refraction was −4.50 D cyl. −5.00 D/140°. For this patient, the postoperative planned target refraction was −1.00 D cyl. −2.00 D/140°. In patients 2 and 3 the postoperative target refraction was plano.

**Figure 4:**
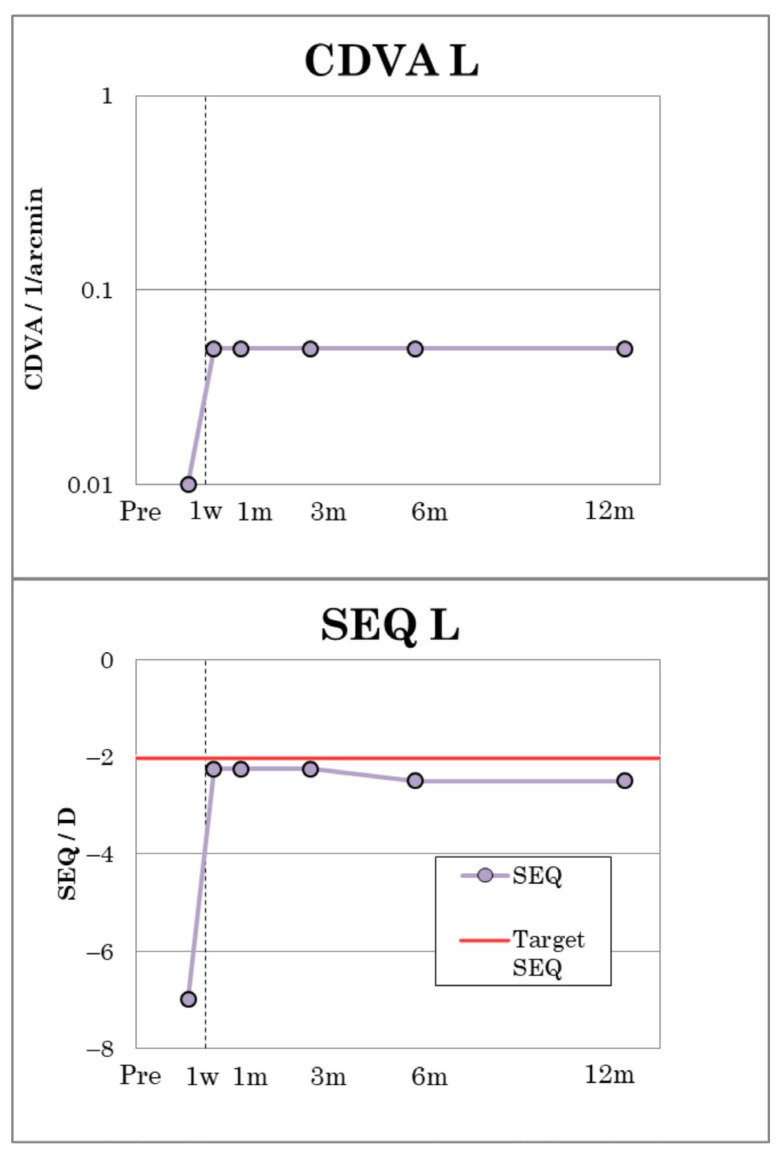
Visual outcomes of patient No. 1.

The amblyopic patient (patient 1) reached a CDVA of 0.05, measured on the Snellen scale, indicating a substantial improvement in visual acuity compared to his preoperative visual performance (Figure 4). The visual outcomes of patient 1 are summarized in Table 3. CDVA remained stable during the entire follow-up period. Postoperatively, patient 1 showed a refraction of −1.00 D cyl. −2.50/130 and an MRSE of −2.25 D, which remained stable up to and including the third postoperative month. After six months, a slight regression to −1.25 D cyl. −2.50/130 with an MRSE of −2.50 D was observable (Figure 4).

**Figure 5:**
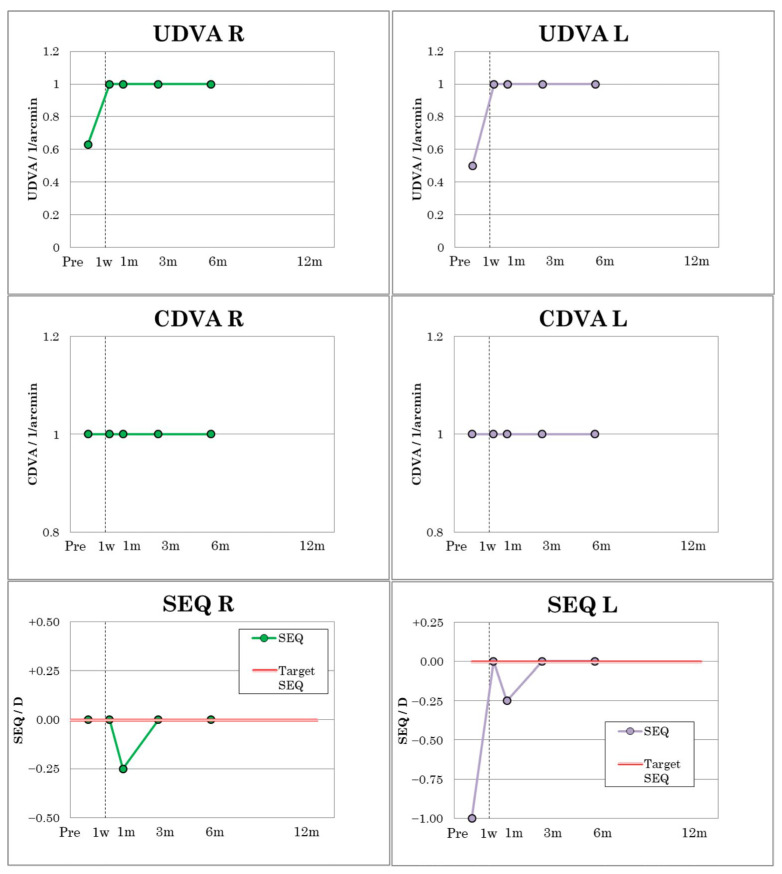
Visual outcomes of patient No. 2.

In patient 2, UDVA and CDVA improved from 0.63 to 1.0 in the right eye from the first postoperative day and from 0.5 to 1.0 in the left eye from the first postoperative week (Figure 5) and remained constant in both eyes until the sixth postoperative month. The visual outcomes of patient 2 are summarized in Table 4. Patient 2 had a cross-cylinder in the right eye with refraction on +0.25 D cyl −0.50/10 and MRSE of 0 D. After one month, a slight myopic shift of −0.25 D in MRSE was observed due to very mild transient dry eye but was no longer present at three- and six-month follow-up (Figure 5).

**Figure 6:**
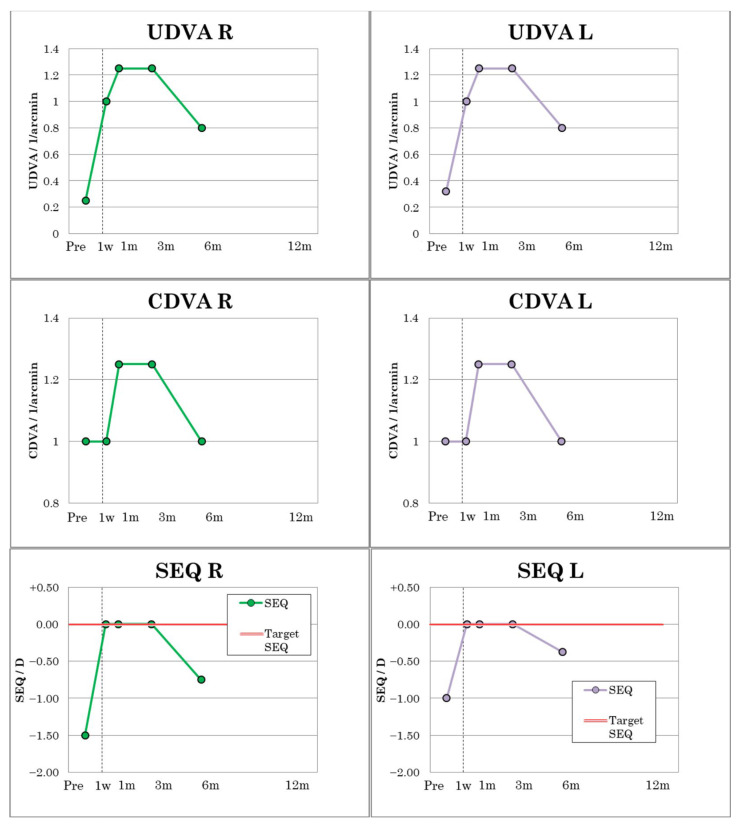
Visual outcomes of patient No. 3.

In patient 3, UDVA and CDVA improved from 0.25 to 1.0 from postoperative day 1 and from 0.32 to 1.0 in the left eye, respectively (Figure 6), and remained constant in both eyes until postoperative month six. However, there were small fluctuations with a UDVA at one month of OU 1.25 and a UDVA at six months of 0.8 in a mild dry eye. The visual outcomes of patient 3 are sumamrized in Table 5. Patient 3 had a preoperative MRSE of −1.50 D in the right eye and −1.00 D in the left. Over the first three postoperative months, the MRSE remained at 0.00 D, but by the sixth postoperative month, a slight regression occurred with MRSE R of −0.75 D and L of −0.375 D (Figure 6), which was due to a transient slight dry eye.

**Figure 7:**
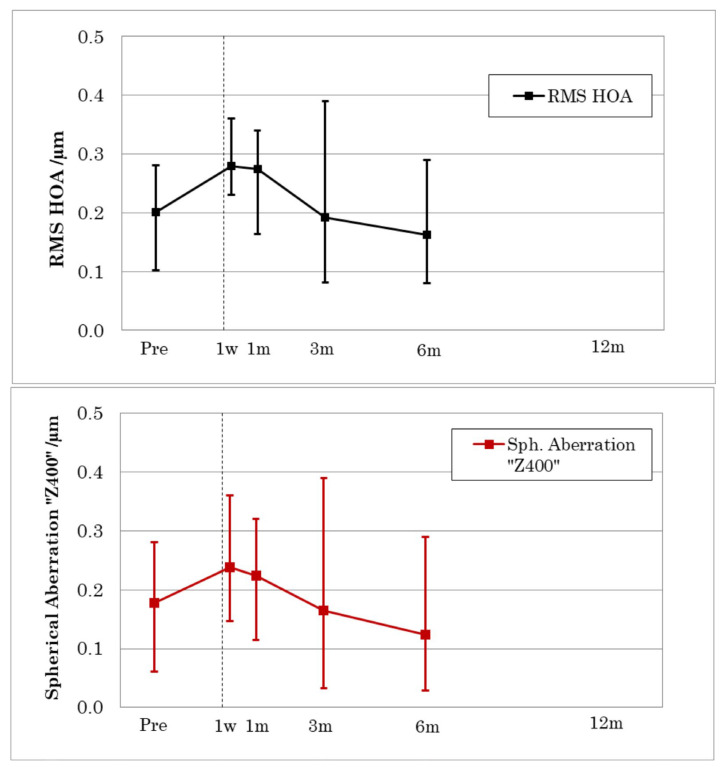
Change of HOA RMS (**top**) and spherical aberration (**bottom**) up to six-month follow-up. Data points represent mean values for all five eyes. Error bars indicate min and max observed values.

In all patients’ eyes, higher-order aberration (HOA) RMS and spherical aberration (Z400) were measured and analyzed over a 6 mm zone up to six-month follow-up. Preoperatively, an average HOA RMS value over all five eyes of 0.20 µm (range 0.10–0.28 µm) was determined. Postoperatively, the average value increased to 0.28 µm (range 0.23–0.36 µm) after the first postoperative week, leveled off at 0.27 µm (range 0.16–0.34 µm) after one month. It decreased to an average of 0.192 µm (range 0.08–0.39 µm) after three months and further to 0.16 µm (range 0.08–0.29 µm) after six months (Figure 7). 

**Figure 8:**
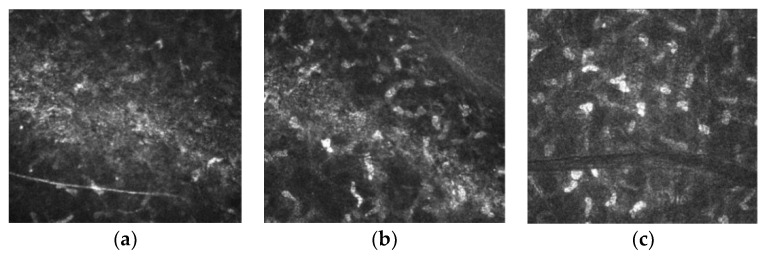
Confocal laser microscopy imaging in the interface area (**a**) three days postoperatively, (**b**) 10 days postoperative, (**c**) one month postoperatively.

Confocal laser microscopy revealed a slight interface edema on the third postoperative day (Figure 8a), which regressed slightly by the tenth postoperative day but was still present (Figure 8b). In the tissue adjacent to the interface oedema, no necrotic changes are observable, and keratocytes seem intact. After one month postoperatively, edema regressed completely, revealing only a visible cut line and completely intact keratocytes. These observations are comparable to those made with recent excimer lasers (Figure 8c).

## 4. Discussion

Within the scope of the case series at hand, the eye which was treated as first using AquariuZ solid-state laser system was a severely amblyopic eye of patient #1, with a CDVA of 0.01. The amblyopia was due to congenital anisometropia and high astigmatism. We aimed to reduce myopic refraction by 3.50 D of sphere and 3.00 D of astigmatism. After six months, we could observe a small undercorrection of 0.25 D for sphere and 0.50 D for astigmatism. However, we were able to observe a clear cornea at all postoperative time points with no haze and no other side effects. Postoperatively, the patient had a CDVA increase of 0.05, which he subjectively considered to be a beneficial gain. Following this initial case, further patients were considered for treatment with the AquariuZ laser. 

In the other two patients, a very good uncorrected visual acuity was observed postoperatively, but also along with a slight refractive regression of 0.25 and 0.5 D, respectively. The slight shift is due to a slight dry eye at the time of measurement. Here, too, no haze could be detected at any time point. Again, the patients were subjectively satisfied with the visual outcome. 

No CDVA lines were lost in any of the operated eyes at any follow-up visit. At six-month follow-up, the safety index of patient 1 was 5, and those for patients 2 and 3 were 1, respectively.

Analysis of the Higher-Order Aberration RMS (HOA) revealed that the applied aspheric optimized profiles did not induce higher-order aberrations nor spherical aberration in these three individual patients. These results are also consistent with recently reported outcomes of induced Higher-Order Aberrations (HOA) and spherical aberration after LASIK in low to moderate myopia [29,30].

Lastly, confocal laser microscopy imaging showed a slight edema present in the interface postoperatively, which resolved after one month. Furthermore, no avital cells were observed in the interface and adjacent tissue. The reported observations, based on the surgeon’s vast refractive experience, closely resemble those seen in the typical LASIK refractive surgery using excimer lasers. 

It should be pointed out that the initially obtained good results described above could be achieved without a nomogram and match the previously reported good safety and efficacy profiles reached with solid-state lasers of other research groups [17,18,24,25,26,27,28].

In addition, previously reported characteristics of solid-state laser sources, such as silent operation and the wet ablation process, suggesting a lower susceptibility to corneal hydration, could be reproduced [24,25,26,27,28]. The wet procedure potentially means less thermal stress and consequently less tensile stress in the irradiated tissue, which as an advantage resulting in a faster healing process [24,31,32].

## 5. Conclusions

The first clinical results with a new solid-state ablation laser are encouraging and underline the potential benefits of this technology. Solid-state lasers at a new performance level are both realizable and capable of meeting expectations in the refractive surgery domain. The presented results demonstrate that with the latest technological advancements, the historically determined developmental gap between excimer lasers and solid-state lasers can finally be overcome.

## Figures and Tables

**Table 1:** Preoperative and postoperative CDVA up to six-month follow-up and safety indices of all patients.

Patient No.	Eye	CDVA Pre	CDVA 1d	CDVA 1W	CDVA 1M	CDVA 3M	CDVA 6M	SI 6M
1	L	0.01	0.01	0.05	0.05	0.05	0.05	5
2	R	1.0	1.0	1.0	1.0	1.0	1.0	1
	L	1.0	0.8	1.0	1.0	1.0	1.0	1
3	R	1.0	0.8	1.0	1.25	1.25	1.0	1
	L	1.0	0.8	1.0	1.25	1.25	1.0	1

**Table 2:** Preoperative UDVA versus postoperative UDVA up to six-month follow-up. HM indicates that UDVA was not measurable on the Snellen scale, yet the patient perceived hand motion at 50 cm distance.

Patient No.	Eye	UDVA Pre	UDVA 1d	UDVA 1W	UDVA 1M	UDVA 3M	UDVA 6M
1	L	HM	HM	HM	HM	HM	HM
2	R	0.63	1.0	1.0	1.0	1.0	1.0
	L	0.5	0.8	1.0	1.0	1.0	1.0
3	R	0.25	1.0	1.0	1.25	1.0	0.8
	L	0.32	1.0	1.0	1.25	1.0	0.8

**Table 3:** Summary of visual outcomes of patient 1.

Patient 1	Eye	Sph/D	Cyl/D	A/°	SEQ/D
PreOp	R	−4.50	−5.00	140	−7.00
Target	R	−1.00	−2.00	140	−2.00
1 Week	R	−1.00	−2.50	130	−2.25
1 Month	R	−1.00	−2.50	130	−2.25
3 Months	R	−1.00	−2.50	130	−2.25
6 Months	R	−1.25	−2.50	130	−2.50

**Table 4:** Summary of visual outcomes of patient 2.

Patient 2	Eye	Sph/D	Cyl/D	A/°	SEQ/D
PreOp	R	+0.25	−0.50	10	0.00
	L	−0.75	−0.50	0	−1.00
Target	R	plano			
	L	plano			
1 Week	R	0.00	0.00	0	0.00
	L	0.00	0.00	0	0.00
1 Month	R	0.00	−0.50	170	−0.25
	L	0.00	−0.50	0	−0.25
3 Months	R	0.00	0.00	0	0.00
	L	0.00	0.00	0	0.00
6 Months	R	0.00	0.00	0	0.00
	L	0.00	0.00	0	0.00

**Table 5:** Summary of visual outcomes of patient 3.

Patient 3	Eye	Sph/D	Cyl/D	A/°	SEQ/D
PreOp	R	−1.25	−0.50	162	−1.50
	L	−0.75	−0.50	10	−1.00
Target	R	plano			
	OS	plano			
1 Week	R	0.00	0.00	0	0.00
	L	0.00	0.00	0	0.00
1 Month	R	0.00	0.00	0	0.00
	L	0.00	0.00	0	0.00
3 Months	R	0.00	0.00	0	0.00
	L	0.00	0.00	0	0.00
6 Months	R	−0.50	−0.50	0	−0.75
	L	−0.25	−0.25	25	−0.375

## Data Availability

The data presented in this study are available on request from the authors, in particular the datasets are archived in the clinics treated. The data are not publicly available as they contain information that could compromise the privacy of the participants.

## References

[1] Houtermans F.G. (1960). Über Massen-Wirkung im optischen Spektralgebiet und die Möglichkeit absolut negativer Absorption für einige Fälle von Molekülspektren (Licht-Lawine). Helv. Phys. Acta.

[2] Basting D., Stamm U. (2001). The Development of Excimer Laser Technology-History and Future Prospects. Z. Für Phys. Chem..

[3] Srinivasan R., Mayne-Banton V. (1982). Self-developing photoetching of poly (ethylene terephthalate) films by far-ultraviolet excimer laser radiation. Appl. Phys. Lett..

[4] Srinivasan R., Leigh W.J. (1982). Ablative photodecomposition: Action of far-ultraviolet (193 nm) laser radiation on poly(ethylene terephthalate) films. J. Am. Chem. Soc..

[5] Trokel S.L., Srinivasan R., Braren B. (1983). Excimer Laser Surgery of the Cornea. Am. J. Ophthalmol..

[6] Seiler T., Bende T., Wollensak J. (1987). Astigmatismuskorrektur mit dem Excimer Laser. Klein Monbl. Augenheilkd..

[7] L’Esperance F.A., Taylor D.M., Warner J.W. (1988). Human Excimer Laser Keratectomy: Short-Term Histopathology. J. Refract. Surg..

[8] Lin J.T. (1990). Non-linear crystals for tunable coherent sources. Opt. Quantum Electron..

[9] Ren Q.S., Gailitis R.P., Tompson K.P., Lin J.T. (1990). Ablation of the cornea and synthetic polymers using a UV (213 nm) solid state laser. IEEE J. Quantum. Electron..

[10] Ren Q.S., Simon G., Parel J.M. (1993). Ultraviolet Solid-state Laser (213-nm) Photorefractive Keratectomy: In Vitro Study. Ophthalmology.

[11] Swinger C., Lai S., Johnson D., Gimbel H., Lai M., Zheng W. (1996). Surface photorefractive keratectomy for correction of hyperopia using the Novatec laser—3 month follow-up. Investig. Ophthalmol. Vis. Sci..

[12] Carr J.D., Hersh P.S. (1996). Excimer laser technology: Key concepts for the ophthalmologist. Semin. Ophthalmol..

[13] Lin J.T. (1995). Critical review on refractive surgical lasers. Opt. Eng..

[14] Stojanovic A., Nitter T.A. (2001). 200 Hz flying-spot technology of the LaserSight LSX excimer laser in the treatment of myopic astigmatism: Six and 12 month outcomes of laser in situ keratomileusis and photorefractive keratectomy. J. Cataract. Refract. Surg..

[15] Anderson I., Sanders D.R., van Saarloos P., Ardrey W.J. (2004). Treatment of irregular astigmatism with a 213 nm solid-state, diode-pumped neodymium:YAG ablative laser. J. Cataract. Refract. Surg..

[16] Roszkowska A.M., Korn G., Lenzner M., Kirsch M., Kittelmann O., Zatonski R., Ferreri P., Ferreri G. (2004). Experimental and clinical investigation of efficiency and ablation profiles of new solid-state deep-ultraviolet laser for vision correction. J. Cataract. Refract. Surg..

[17] Tikhov A.V., Kuznetsov D.V., Tikhov A.O., Tikhova E.V. (2014). Analysis of the clinical results of refractive operations performed on the domestic solid-state laser system “OLIMP-2000/213-300Hz”. Mod. Technol. Cataract. Refract. Surg..

[18] Tikhov A.V., Kuznetsov D.V., Tikhov A.O., Tikhova E. (2015). Analysis of two-year clinical observations of the results of 2200 operations performed on the domestic solid-state refractive laser system “OLIMP-2000/213-300Hz”. Mod. Technol. Cataract. Refract. Surg..

[19] Hale G.M., Querry M.R. (1973). Optical Constants of Water in the 200-nm to 200-μm Wavelength Region. Appl. Opt..

[20] Dair G.T., Ashman R.A., Eikelboom R.H., Reinholz F., Van Saarloos P.P. (2001). Absorption of 193- and 213-nm laser wavelengths in sodium chloride solution and balanced salt solution. Arch. Ophthalmol..

[21] Dougherty P.J., Wellish K.L., Maloney R.K. (1994). Excimer laser ablation rate and corneal hydration. Am. J. Ophthalmol..

[22] Kim W.S., Jo J.M. (2001). Corneal hydration affects ablation during laser in situ keratomileusis surgery. Cornea.

[23] Schallhorn S.C., Amesbury E.C., Tanzer D.J. (2006). Avoidance, Recognition, and Management of LASIK Complications. Am. J. Ophthalmol..

[24] Shah S., Sheppard A.L., Castle J., Baker D., Buckhurst P.J., Naroo S.A., Davies L.N., Wolffsohn J.S. (2012). Refractive outcomes of laser-assisted subepithelial keratectomy for myopia, hyperopia, and astigmatism using a 213 nm wavelength solid-state laser. J. Cataract. Refract. Surg..

[25] Ng-Darjuan M.F., Evangelista R.P., Agahan A.L.D. (2013). Photorefractive Keratectomy with Adjunctive Mitomycin C for Residual Error after Laser-Assisted In Situ Keratomileusis Using the Pulzar 213 nm Solid-State Laser: Early Results. ISRN Ophthalmol..

[26] Felipe A.F., Agahan A.L.D., Cham T.L., Evangelista R.P. (2011). Photorefractive keratectomy using a 213 nm wavelength solid-state laser in eyes with previous conductive keratoplasty to treat presbyopia: Early results. J. Cataract. Refract. Surg..

[27] Vengris M., Gabryte E., Aleknavicius A., Barkauskas M., Ruksenas O., Vaiceliunaite A., Danielius R. (2010). Corneal shaping and ablation of transparent media by femtosecond pulses in deep ultraviolet range. J. Cataract. Refract. Surg..

[28] Piñero D.P., Ribera D., Pérez-Cambrodí R.J., Ruiz-Fortes P., Blanes-Mompó F.J., Alzamora-Rodríguez A., Artola A. (2014). Influence of the difference between corneal and refractive astigmatism on LASIK outcomes using solid-state technology. Cornea.

[29] Russo A., Filini O., Salvalai C., Boldini A., Festa G., Delcassi L., Morescalchi F., Semeraro F. (2021). Two-Year Changes in Corneal Spherical Aberration After Laser-Assisted In Situ Keratomileusis and Photorefractive Keratectomy in Regular and Wavefront-Guided Ablations. Ophthalmol. Ther..

[30] Agarwal S., Thornell E., Hodge C., Sutton G., Hughes P. (2018). Visual Outcomes and Higher Order Aberrations Following LASIK on Eyes with Low Myopia and Astigmatism. Open Ophthalmol. J..

[31] Roszkowska A.M., De Grazia L., Ferreri P., Ferreri G. (2006). One-year clinical results of photorefractive keratectomy with a solid-state laser for refractive surgery. J. Refract. Surg..

[32] Tsiklis N.S., Kymionis G.D., Kounis G.A., Pallikaris A.I., Diakonis V.F., Charisis S., Markomanolakis M.M., Pallikaris I.G. (2007). One-year results of photorefractive keratectomy and laser in situ keratomileusis for myopia using a 213 nm wavelength solid-state laser. J. Cataract. Refract. Surg..

